# Role of open cerclage wiring in patients with comminuted fractures of the femoral shaft treated with intramedullary nails

**DOI:** 10.1186/s13018-021-02633-w

**Published:** 2021-08-07

**Authors:** Tzu-Hao Wang, Hao-Chun Chuang, Fa-Chuan Kuan, Chih-Kai Hong, Ming-Long Yeh, Wei-Ren Su, Kai-Lan Hsu

**Affiliations:** 1 Department of Orthopaedics, Kaohsiung Municipal Min-Sheng Hospital, Kaohsiung, Taiwan, R.O.C.; 2 Department of Orthopaedic Surgery, National Cheng Kung University Hospital, College of Medicine, National Cheng Kung University, Tainan, Taiwan, R.O.C.; 3 Department of Biomedical Engineering, National Cheng Kung University, Tainan, Taiwan, R.O.C.; 4 Division of Traumatology, National Cheng Kung University Medical Center, Tainan, Taiwan, R.O.C.; 5 Skeleton Materials and Bio-compatibility Core Lab, Research Center of Clinical Medicine, National Cheng Kung University Hospital, College of Medicine, National Cheng Kung, Tainan, Taiwan, R.O.C.

## Abstract

**Introduction:**

The role of open cerclage wiring in comminuted femoral shaft fracture treatment with intramedullary nails remains unclear. Here, we analyzed the effect of open cerclage wiring and the risk factors for nonunion after interlocking nailing in comminuted femoral shaft fracture treatment. We hypothesized that open cerclage wiring can be applied in patients with severe comminuted femoral shaft fractures without affecting bone healing.

**Patients and methods:**

This retrospective cohort study used data from consecutive patients who underwent interlocking nail fixation of a comminuted femoral shaft fracture between January 1, 2009, and December 31, 2016. First, eligible patients were divided into the wire and no wire groups according to the surgical technique used, and their union rate was recorded. The patients were then divided into the union and nonunion groups, and their perioperative data were analyzed.

**Results:**

In total, 71 comminuted femoral shaft fractures treated with interlocking nail fixation were included: 38 fractures (53.5%) augmented with the open wiring technique and 33 reduced with closed or mini-open techniques without wiring. The wire group demonstrated significant improvements in fracture reduction compared with the no wire group, whereas no significant difference was observed in the union rate between the wire and no wire groups (*p* = 0.180). Moreover, 46 (65%) of 71 fractures achieved union smoothly, and no significant difference was observed in any perioperative data between the union and nonunion groups.

**Discussion:**

Augmentation with open cerclage wiring is indicated for comminuted femoral shaft fractures treated with intramedullary nails, even when the fragments are large or greatly displaced. Thus, open cerclage wiring can be used for fracture treatment without decreasing the union rate.

## Introduction

Although interlocking nailing is a preferred procedure for treating femoral shaft fractures and has a high union rate [1], fracture comminution leads to challenges. Anatomically restoring multiple displaced fragments is difficult [2]. Thus, to facilitate reduction and increase stability, cerclage wires are widely used. In 1987, Fitzgerald documented excellent results of using cerclage wires in the treatment of comminuted fractures of the femur [3]. Subsequently, successful procedures for treating comminuted fractures of the femur have been reported [4–9], and percutaneous wiring techniques are being continually improved [10, 11].

However, fractures reported in the literature have mainly fractures of the peritrochanteric area [4–8], not of the shaft. Unlike peritrochanteric fractures, femoral shaft fractures are usually caused by high-energy trauma. The greatly displaced fragments in such fractures [12, 13] usually make percutaneous wiring impossible. Surgeons have to perform open reduction and internal fixation using one or more cerclage wires combined with an intramedullary nails to achieve stability in the treatment of comminuted fractures of the femoral shaft. However, open cerclage wiring has some drawbacks. To achieve anatomical reduction, some soft tissues have to be detached from fragments, which may disturb the blood supply and result in nonunion, increasing the infection rate [14, 15]. Moreover, the passing cerclage wire may strangulate large blood vessels [16, 17] and periosteal blood vessels [18], which may affect bone healing.

Surgeons may, however, attempt to reduce fragments using an all-closed or mini-open method to preserve soft tissue coverage. However, performing these techniques is difficult, and a residual gap may occur after reduction, increasing the nonunion risk [12]. Moreover, “bridging” interlocking nails through the comminuted region of the femoral shaft do not provide sufficient stability. Therefore, this study analyzed the timing and effect of open cerclage wire use and identified factors affecting bone union when treating comminuted femoral shaft fractures. We hypothesized that open cerclage wiring can be applied in patients with severe comminuted fractures of the femoral shaft without affecting bone healing.

## Materials and methods

### Participants

This retrospective cohort study was approved by the institutional review board, and patients who underwent surgical fixation of a femoral fracture at National Cheng Kung University Hospital and its Douliu branch between January 1, 2009, and December 31, 2016, were recruited. All fractures were identified using the coding system of the National Health Insurance database in Taiwan. The inclusion criterion was a comminuted femoral shaft fracture, namely, an Arbeitsgemeinschaft für Osteosynthesefragen or Orthopaedic Trauma Association (AO/OTA) class 32-B or 32-C femoral fracture. Femoral shaft fractures were defined as being between 5 cm distal to the lesser tuberosity and 9 cm proximal to the knee joint line [19]; fractures that extended beyond this range were excluded. The exclusion criteria were simple fractures (AO/OTA class 32-A), fractures in skeletally immature patients, revision procedures, peri-implant fractures, open fractures classified as Gustilo III, pathological fractures, fractures fixed using other implants, and insufficient follow-up (less than 24 months). Each extremity undergoing a surgical procedure was evaluated independently. Thus, patients with bilateral femoral shaft fractures were regarded as two independent participants.

The primary aim of the study was to determine the effect of open cerclage wire use. Eligible patients were categorized according to the surgical technique used. The wire group comprised fractures fixed using interlocking nails and augmented with the open wiring technique. The no wire group comprised fractures fixed using only interlocking nails through closed or mini-open reduction. After grouping, the medical records of each patient and fractures were reviewed to obtain patient demographic characteristics, including age, sex, fracture classification (AO/OTA), largest fragment size, fragment number, and displacement of proximal and distal fragments. Only fragments measuring more than 10 mm were counted. Images of patients included in this study were displayed and measured using digital imaging and communication in medicine image-viewing software (πView^TM^, INFINITT Co., Ltd., Seoul, Korea).

### Surgical method and postoperative rehabilitation

**Fig. 1:**
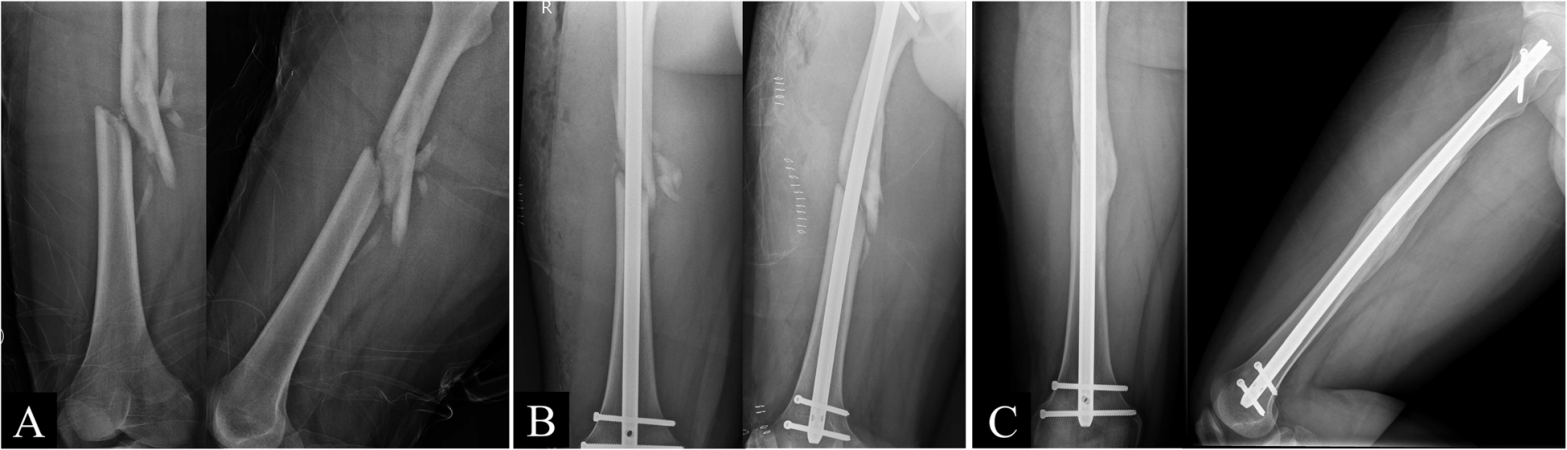
(**A**) Preoperative, (**B**) postoperative, and (**C**) 15-month postoperative images of a 19-year-old man with a comminuted fracture of the right femur from a motorcycle accident. Reduction and fixation were achieved via a mini-open technique, and the fracture healed 15 months after the surgery

**Fig. 2:**
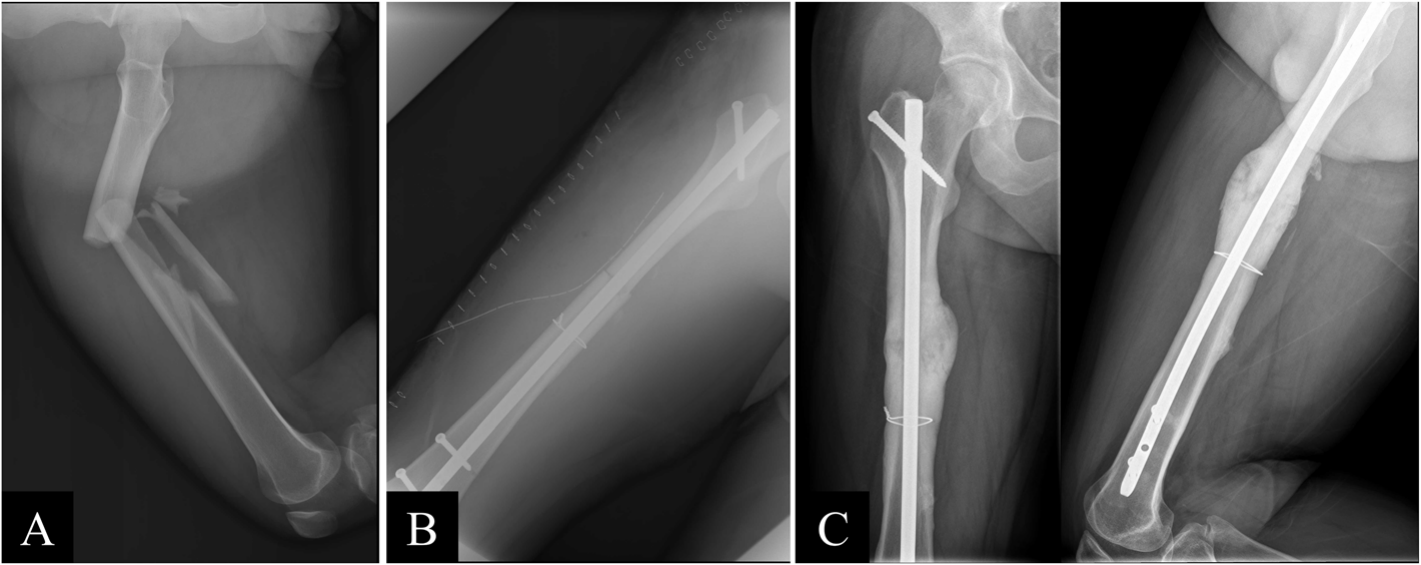
(**A**) Preoperative, (**B**) postoperative, and (**C**) 14-month postoperative images of a 24-year-old man with a comminuted fracture of the right femur from a traffic accident. Reduction was successfully achieved using open cerclage wiring, and the fracture healed 14 months after the surgery

This study focused on the effect of open cerclage wiring; therefore, the intraoperative details of the patient position and the nail insertion technique were not considered. The patient may have been treated on a fracture table or in a lateral decubitus position. The interlocking nails could have been inserted in an antegrade manner from the piriformis fossa or greater trochanter or inserted in a retrograde manner. In the no wire group, the fracture was reduced through traction, manipulation, or use of a bone hook and joystick in the mini-open technique (Fig. 1). In contrast, in the wire group, the fracture was reduced using a bone holder or reduction clamp directly, and simple stainless cerclage wires were passed through a cannulated semicircular wire passer (Fig. 2).

Postoperative rehabilitation programs were similar for all individuals. Partial weight bearing under crutch protection was allowed immediately after surgery except if there was a concomitant fracture of the contralateral lower limb or pelvis. The timing of full weight bearing depended on callus formation and was usually 6 to 8 weeks after the surgery, when some callus formation was noted on the plain film.

### Data collection

After the surgery, the displacement of proximal and distal fragments was measured to evaluate reduction quality, and the modified radiographic union for tibial fractures (mRUST) score [20, 21] was used to assess bone healing. The mRUST score was evaluated at 1, 3, 6, 9, and 12 months after surgery. To avoid observation bias, two authors evaluated the outcomes independently. Disagreements were resolved through discussion with a third author.

The outcomes of the study included the rate of fracture union and nail breakage. Considering that the included fractures were comminuted, “union” was defined as an mRUST score equal to or greater than 13 within 24 months postoperatively [20, 21]. In contrast, “nonunion” was defined as an mRUST score of less than 13, or the need for any revision procedure, including nail exchange, plate augmentation, or bone grafting, within 24 months postoperatively.

Regarding the second aim of the study, the patients were then assigned to the union and nonunion groups, and factors that may affect bone healing, including age, smoking, body mass index (BMI), brain injury, degree of preoperative and postoperative fracture comminution, and open cerclage wire use, were analyzed. Brain injury was defined as a decrease in the Glasgow Coma Scale score compared with the initial score and any evidence of intracranial hemorrhage or brain edema on computed tomography.

### Statistical analysis

The results were analyzed using the SPSS statistical software (SPSS, Inc., USA). The chi-square test was conducted to evaluate categorical variables, such as the union rate. Continuous variables, including the size and displacement of fragments, were evaluated using unpaired Student’s *t* test. A *p* value of less than 0.05 was considered statistically significant.

## Results

**Fig. 3:**
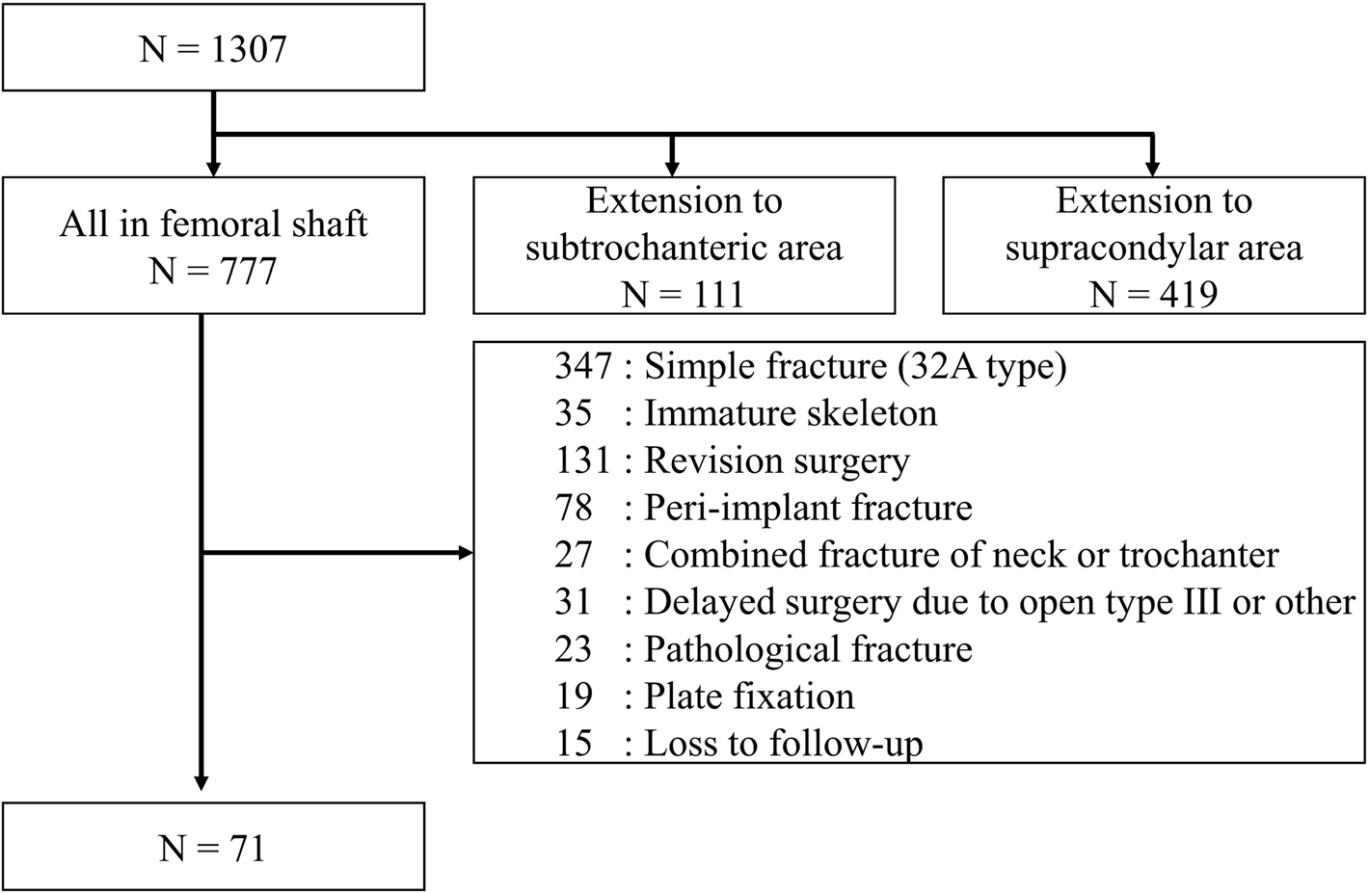
Patient recruitment flowchart

Using the coding system of the National Health Insurance database in Taiwan, 71 fractures treated with interlocking nail fixation were included in this study, namely, 38 fractures (53.5%) augmented with the open wiring technique and the remaining 33 fractures reduced with a closed or mini-open technique without wiring. The patient flowchart is listed in Fig. 3.

Of the 71 fractures, 45 were in men (63.4%) and 26 were in women (36.6%); the mean age of the patients (± standard deviation) was 28.5 ± 13.4 years, and no significant differences were observed in the age or sex distribution between the wire and no wire groups. In total, 43 fractures were on the right side (60.6%), and the other 28 were on the left side (39.4%), with a similar distribution between the two groups (Table 1).

**Table 1:** Demographics of patients with a comminuted femoral shaft fracture treated using an interlocking nail with and without an open cerclage wire

Characteristic	Wire (*n* = 38)	No wire (*n* = 33)	*p* value
**Age**	27.74 ± 14.77	29.30 ± 12.09	0.635
**Sex**			
Male	25	20	0.651
Female	13	13	
**Side**			0.847
Right	23	20	
Left	15	13	

Considering the preoperative severity of the fractures, the wire group showed significantly more severe fractures than the no wire group in terms of the fragment number (*p* = 0.043), largest fragment size (*p* = 0.004), proximal fragment displacement (*p* = 0.006), distal fragment displacement (*p* = 0.009), and Winquist and Hansen classification (*p* < 0.001). In contrast, the wire group showed significantly less displacement (*p* < 0.001) proximally and distally than the no wire group postoperatively (Table 2).

**Table 2:** Fracture pattern and union rate in patients with a comminuted femoral shaft fracture treated using an interlocking nail with and without an open cerclage wire

Characteristic	Wire (*n* = 38)	No wire (*n* = 33)	*p* value
**Classification**			0.06
32B	31	32	
32C	7	1	
**Fragment number**	1.53 ± 0.79	1.24 ± 0.49	**0.043**
**Fragment size (mm)**	87.66 ± 30.40	66.09 ± 29.03	**0.004**
**Proximal displacement (mm)**			
Preoperative	21.64 ± 16.94	12.80 ± 7.50	**0.006**
Postoperative	2.96 ± 1.88	7.51 ± 3.32	**< 0.001**
**Distal displacement (mm)**			
Preoperative	20.08 ± 13.52	13.05 ± 7.50	**0.009**
Postoperative	3.57 ± 2.91	7.11 ± 3.26	**< 0.001**
**Union within 24 months**	22	24	0.180
**Nail breakage**	6	4	0.558

Considering the postoperative results, 46 of 71 fractures achieved union smoothly, for a union rate of 65%. No significant difference existed in the union rate between the wire and no wire groups (*p* = 0.180) (Table 2). Among the united fractures, the average union time was 11.77 months in the wire group and 10.29 months in the no wire group, with no significant difference (*p* = 0.206). Nail breakage occurred in 10 fractures, and the average duration from surgery to nail breakage was 11.4 months (Table 2).

Regarding the factors affecting fracture union, no significant difference was observed between the union and nonunion groups regarding age, smoking, BMI, brain injury, degree of preoperative and postoperative fracture comminution, or open cerclage wire use (Table 3).

**Table 3:** Patient demographics and fracture pattern and union rate in patients with a comminuted femoral shaft fracture treated using an interlocking nail with and without bone union

Characteristic	Union (*n* = 47)	Nonunion (*n* = 24)	*p* value
**Age**	27.85 ± 13.69	29.67 ± 12.81	0.596
**Sex**			0.352
Male	28	17	
Female	19	7	
**Smoking**	11	6	0.882
**BMI**	23.00 ± 4.65	24.73 ± 4.14	0.140
**Open fracture**	8	4	0.970
**Brain injury**	5	6	0.113
**Bilateral femur involvement**	5	1	0.354
**Classification**			0.576
32B	41	22	
32C	6	2	
**Fragment number**	1.47 ± 0.74	1.25 ± 0.52	0.207
**Largest fragment size (mm)**	76.05 ± 32.60	75.85 ± 29.46	0.560
**Proximal displacement (mm)**			
Preoperative	16.52 ± 12.20	19.51 ± 17.07	0.404
Postoperative	5.05 ± 3.11	5.23 ± 4.08	0.841
**Distal displacement (mm)**			
Preoperative	15.80 ± 9.47	18.78 ± 14.89	0.316
Postoperative	5.56 ± 3.15	4.55 ± 4.09	0.259
**Nail size**	11.13 ± 1.06	11.48 ± 1.02	0.202

## Discussion

The application of interlocking nails in comminuted fractures of the femoral shaft is challenging for orthopedic surgeons. Greatly displaced fragments make closed reduction impossible, even if a percutaneous wire passer or bone hook is used through a mini-open technique. Thus, open cerclage wiring is the preferred method for achieving and maintaining reduction for further reaming and nail application. However, open cerclage wires involve the risk of disrupting soft tissue and periosteal blood flow. This study aimed to identify the role of augmentation with cerclage wiring in patients with comminuted fractures of the femoral shaft treated with intramedullary nails, especially in terms of the timing, effect, and drawbacks.

The first result of the study is that augmentation with open cerclage wiring was usually used in the treatment of severe comminuted fractures and effectively achieved fracture reduction. Thus, cerclage wires could be used not only for reducing long spiral and torsional fractures [22, 23] but also for effectively treating severe comminuted fractures with greatly displaced fragments (Fig. 2). Moreover, Scharf et al. [24] reported superior fixation stability with a combination of cerclage wiring and interlocking nailing in a femoral fracture model. Therefore, cerclage wiring could both simplify the surgery and improve the stability of fixation.

The second result is that the open cerclage wiring technique did not decrease the union rate or union time of comminuted femoral shaft fractures. In the past, open cerclage wiring has been criticized for causing extensive soft tissue dissection during the approach to the fracture site; thus, there has been a dilemma regarding whether to preserve the soft tissue or to obtain improved reduction. However, the literature [25–27] has failed to document a significant difference between open and closed nailing for the treatment of femoral shaft fractures. Furthermore, using a direct open approach, surgeons can directly observe the fracture line to bring the fragments together and to achieve anatomical reduction with less fluoroscopy exposure [26]. The presented results suggest that open nailing for the treatment of comminuted femoral shaft fractures would not disturb callus formation or bone healing.

Another concern regarding the use of open cerclage wiring is the risk of cerclage wires disrupting the blood supply. The blood supply may be disrupted through the strangulation of blood vessels [28, 29] and contact between the cerclage wires and the bone surface. The risk of vascular injury due to the cerclage passer has been reported as 1.59% in proximal femoral shaft fractures and 7.14% in distal femoral shaft fractures [28]. However, previously reported cases of vascular injury mostly occurred due to the percutaneous cerclage wiring technique. With the percutaneous technique, it is difficult to ensure that the cerclage passer is passed sufficiently subperiosteally and that the vessel is outside the cerclage loop. However, by using the open wiring technique, surgeons are able to ensure that the wire is placed subperiosteally and thus prevent vessel strangulation.

Furthermore, the contact between the cerclage wire and bone surface impedes the blood supply, especially when the endosteal blood supply might have been disrupted by reaming and the application of interlocking nails. However, the histological and anatomical study of femoral vascularity by Pazzaglia et al. [30] suggested that the periosteal vascular supply is circumferential, rather than longitudinal, with multiple musculo-periosteal vessels nourishing the periosteal layer. Moreover, Apivatthakakul et al. [17] and Kennedy et al. [9] proved that cerclage wiring resulted in minimal disruption of the femoral blood supply despite the location of the cerclage wire and the distance between the wire loops.

Moreover, our results suggest that cerclage wiring is beneficial in treating severe comminuted fractures of the femoral shaft. Lin et al. [12] and Lee et al. [13] observed that a large fragment size and great distance of fragment displacement in comminuted femoral shaft fractures were associated with nonunion. In our study, the wire group had significantly larger fragment sizes and greater distances of fragment displacement than the no wire group; thus, it theoretically should have shown an inferior union rate. However, these two groups showed a similar union rate. The benefits of anatomical reduction and augmented fixation seem to outweigh the disadvantages of extensive soft tissue and periosteal blood disruption. Although this is not a robust statistical analysis, the result suggests that cerclage wiring is safe and might be beneficial.

Regarding the final result of the study, we failed to identify risk factors for nonunion, including smoking, BMI, and preoperative and postoperative fragment number and displacement. The risk factors for nonunion in femoral shaft fracture treatment with interlocking nails remains controversial. Taitsman et al. [31] found that open fractures, tobacco use, and delayed weight bearing are risk factors for femoral nonunion. In contrast, Metsemakers et al. [14], Lin et al. [12], and Lee et al. [13] documented that the severity of fracture comminution, including the AO/OTA classification and degree of preoperative fragment displacement, was the only risk factor for nonunion. The eligible fractures in our study were all comminuted (AO/OTA class 32B/32C) and theoretically had a large degree of fragment displacement, which could explain why the union rate in our study (65%) was lower than that reported previously [1]. Moreover, no significant difference was observed in the union rate or preoperative fragment size or displacement between the groups. This might be explained by the decrease in fracture gaps after cerclage wiring improving fracture healing.

This study has some limitations. First, we attempted to obtain samples with similar fracture patterns in the two groups by excluding simple fractures, but we failed. Thus, we were unable to obtain an objective conclusion from the presented results. Second, data were collected retrospectively, and surgeries were performed by different surgeons. Individual surgeons may follow different indications for the use of interlocking nailing or plating, using cerclage or not, mini-open reduction or closed reduction, and the lateral decubitus position or supine position when treating fractures in the operating room. The postoperative rehabilitation programs of different surgeons were also slightly different, which may have affected the study outcomes, particularly fixation failure. We identified some possible confounding variables, such as the fracture classification. However, some related factors that could not be controlled for, such as bone quality or patient compliance, may have also had an influence. Third, the nonunion rate of 35% is relatively high when compared with previous literature. The reason may cause by that only partial weight bearing was allowed immediately after surgery. Besides, even “minimally invasive” reduction may lead to disruption of the blood supply to fragments. In particular, in this retrospective study with different surgeons, there were no measurements of actual “reduction maneuvers” or soft tissue stripping. Forth, the sample size was relatively small, and the study may have lacked sufficient power to detect meaningful differences between group and the risk factors for nonunion. Finally, this study primarily focused on radiological results and a simple review of medical records. Further investigation is required, and precise measurements of functional results, such as the range of motion, knee score, or score of an injury-specific questionnaire, should be used in future research.

In conclusion, augmentation with open cerclage wiring is indicated in comminuted fractures of the femoral shaft treated with intramedullary nails even when the fragments are large or greatly displaced. By using the open cerclage wiring technique, fractures can be reduced better in radiographic appearance. We advocate that augmentation with open cerclage wiring is safe in comminuted femoral shaft fractures treated with interlocking nails. However, surgeon should be aware of that cerclage wire may add extra hardware to remove if needed in the future, and overgrowth or breakage can make this more difficult.

## Data Availability

The datasets used and/or analyzed during the current study are available from the corresponding author on reasonable request.

## Abbreviations

AO/OTA: Arbeitsgemeinschaft für Osteosynthesefragen or Orthopaedic Trauma Association
mRUST: Modified radiographic union for tibial fractures
BMI: Body mass index
